# Collaborative Autonomous Driving—A Survey of Solution Approaches and Future Challenges

**DOI:** 10.3390/s21113783

**Published:** 2021-05-29

**Authors:** Sumbal Malik, Manzoor Ahmed Khan, Hesham El-Sayed

**Affiliations:** 1 College of Information Technology, United Arab Emirates University, Al Ain, Abu Dhabi 15551, United Arab Emirates; 201990107@uaeu.ac.ae (S.M.); manzoor-khan@uaeu.ac.ae (M.A.K.); 2 Emirates Center for Mobility Research (ECMR), United Arab Emirates University, Al Ain, Abu Dhabi 15551, United Arab Emirates

**Keywords:** cooperative driving, collaboration, lane change, platooning, leader election

## Abstract

Sooner than expected, roads will be populated with a plethora of connected and autonomous vehicles serving diverse mobility needs. Rather than being stand-alone, vehicles will be required to cooperate and coordinate with each other, referred to as cooperative driving executing the mobility tasks properly. Cooperative driving leverages Vehicle to Vehicle (V2V) and Vehicle to Infrastructure (V2I) communication technologies aiming to carry out cooperative functionalities: (i) cooperative sensing and (ii) cooperative maneuvering. To better equip the readers with background knowledge on the topic, we firstly provide the detailed taxonomy section describing the underlying concepts and various aspects of cooperation in cooperative driving. In this survey, we review the current solution approaches in cooperation for autonomous vehicles, based on various cooperative driving applications, i.e., smart car parking, lane change and merge, intersection management, and platooning. The role and functionality of such cooperation become more crucial in platooning use-cases, which is why we also focus on providing more details of platooning use-cases and focus on one of the challenges, electing a leader in high-level platooning. Following, we highlight a crucial range of research gaps and open challenges that need to be addressed before cooperative autonomous vehicles hit the roads. We believe that this survey will assist the researchers in better understanding vehicular cooperation, its various scenarios, solution approaches, and challenges.

## 1. Introduction

Transportation systems all around the world experience different traffic problems, such as increased traffic flow, poor safety, and road accidents. According to the 2019 Urban Mobility report [1], traffic congestion resulted in Americans traveling an extra 8.8 billion h, consuming an extra 3.3 billion gallons of fuel leading to an estimated cost of $166 billion. To cope up with issues, the general idea is to build additional highways, which is impractical now, as most cosmopolitan cities are running out of land. A replacement to this option which has attained significant popularity is to broaden the capacity of existing roads by leveraging the intelligent transportation system (ITS) infrastructure and its sub-components [2]. It is evident from the research that ITS can maximize the capacity of roads, up to 20% [3]. The important component of ITS is collaborative driving (CD), also known as cooperative driving. In this paper, we use both terms interchangeably.

The advancement to simple Cruise Control (CC) has been implemented as Adaptive Cruise Control (ACC). The ACC controller is capable of automatically adjusting the speed of the vehicle by maintaining a constant distance with the following vehicle. It exploits the on-board sensor information, such as from radar or camera and allows the vehicle to brake when it detects that one vehicle is approaching another vehicle ahead. Recently, the Cooperative Adaptive Cruise Control (CACC) has been implemented, which leverages the vehicle to vehicle (V2V) wireless communication system and lets the vehicles share maneuvering information, cooperate, and enhance the autonomy. Grouping vehicles equipped with CACC technology can form a network of collaborative vehicles, referred to as a collaborative driving system.

The collaborative driving system (CDS), the study of inter-vehicle communication and cooperative driving, is one of the growing research areas of the intelligent transportation system. It makes use of inter-vehicle communication (IVC) aiming to share on-board sensor information with the neighboring cooperative vehicles to let them travel through urban traffic and make optimal joint decisions [4]. Vehicles driving in a cooperative environment usually execute the follow, merge, split, and change lane maneuvers to maintain the safety and efficiency of road traffic.

It is worth highlighting here that the notion of collaborative driving may correspond to different decision-making instances and mechanisms in different use cases of autonomous driving. This is to say, in some cases, the decision of collaboration is straightforward, where the vehicles must exchange information. However, in some cases, the decision of collaboration is followed by other decision instances, e.g., in platooning.

Now that the autonomous driving paradigm has swiftly transferred to a more mature stage, we believe that the research community and industry should clearly distinguish between the collaborative decision mechanism. We make an effort in this paper to address the aforementioned issue and walk the readers through the most commonly known autonomous driving decision paradigm by providing a survey of various applications of cooperative driving (car parking, lane change and merge, cooperative intersection management, etc.) focusing on platooning and their available solution approaches, highlighting the gaps, and discussing future challenges of cooperative autonomous driving.

### Contribution

This survey aims to provide background and review of the recent research studies in collaborative driving. The overarching contributions of this paper are summarized as follows:

- Provides a tutorial to assist readers with the background of collaborative autonomous driving and associated concepts.
- Investigates the classification of cooperating driving motivations, types, degrees, and control strategies.
- Devises a taxonomy of collaborative driving by investigating various factors.
- Carries out a literature review on various collaborative driving applications: smart car parking, lane change and merge, and cooperative intersection management aiming, in order to provide a clear understanding of contributed research solution approaches and challenges of these areas.
- A specialized version of collaborative autonomous driving, platooning of higher autonomous levels, has gained huge attention recently. Considering the importance of this application, the paper devotes a whole section to the platooning, its special use-case leader election, various scenarios of electing a leader, and relevant solutions.
- Highlights and categorizes the current open research challenges of platoon leader election that hinder the real-time election of a suitable leader for a platoon.
- Provides a range of crucial research gaps, challenges, and future directions to achieve the goals of cooperative driving.

Based on the scope of this article, only the most relevant articles on collaborative autonomous are considered for this paper. Various prominent sources, such as IEEE Xplore, ACM Digital Library, Springer, and Science Direct, are explored to obtain the most accurate and up-to-date information. Furthermore, various keywords (such as cooperative autonomous driving, collaborative driving, coordination of autonomous vehicles, cooperative maneuvers, platooning, cooperative intersection management, and smart parking) are used to obtain the relevant article for this study. Successive searches are also included to adjust the keywords to retrieve relevant studies. Pertinent data is extracted from all gathered articles, and results are presented after proper analysis. It is ensured that this survey does not become another systematic review article; hence, key proposals are discussed to provide new in-depth information on collaborative driving, its applications, and challenges to provide a sound base for future research works.

This paper is divided into three main parts, namely: (i) taxonomy of collaborative driving, which aims to equip the readers with the background information needed to comprehend the contents of this survey; (ii) analysis of the research literature, which provides a comprehensive survey of the research literature related to the cooperative driving applications; and (iii) challenges and future recommendations, which outlines the challenges of cooperative driving and suggests future directions to motivate more innovative research in this area.

## 2. Taxonomy of Collaborative Driving

**Figure 1:**
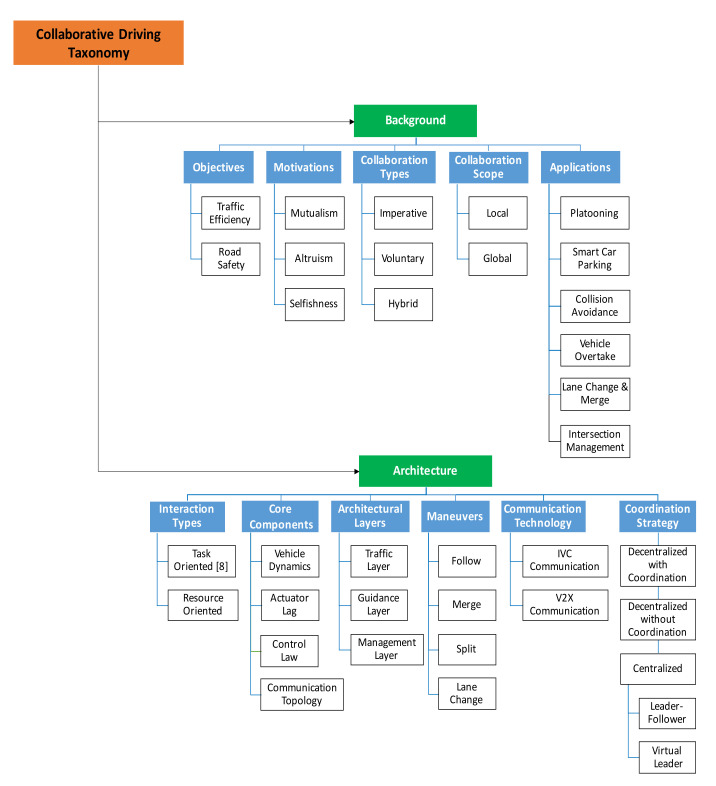
Collaborative driving system (CDS) taxonomy.

This section focuses on equipping the readers with the background information related to collaborative driving. To the best of our knowledge, this paper takes the first step towards summarizing and devising a detailed taxonomy of collaborative autonomous driving (based on available researches), as shown in Figure 1. As illustrated in Figure 1, the proposed taxonomy is divided into two categories:

- *Background Category*—comprised of objectives, motivations, collaboration types, collaboration scopes, and applications.
- *Architecture Category*—consists of interaction types, core components, architectural layers, maneuvers, communication technologies, and coordination strategies.

The tutorial and background information on collaborative driving is very scattered in the literature. Considering this limitation, we propose this taxonomy by carefully choosing the main factors aiming to give a broad overview of collaborative driving to the readers. The goal of the first category is to provide the basic information related to the CDS, whereas the latter part discusses the CDS in more detail, such as the architecture and coordination strategies of CDS. Each component is further discussed in detail in this section.

### 2.1. Collaborative Driving

Collaborative driving, a crucial sub-component of ITS, strives to create automated vehicles seeking to cooperate and navigate through highways using communication technologies. It then adopts a desirable control law to achieve common objectives. To achieve this, the CDS environment should consider four crucial components: (i) *Vehicle Dynamics:* defines the vehicle dynamics (i.e., longitudinal and lateral) with a complete vehicle model; (ii) *Actuator Lag:* describes the information to be exchanged between the vehicles; (iii) *Communication Topology:* determines the connectivity structure of the vehicular network to be used; (iv) *Control Law:* defines the control law to be implemented on each vehicle. The U.S. National Highway Traffic Safety Administration (NHTSA) [5] categorized driving automation into six levels, and collaborative driving can exist at different levels of automation. As the automation level increases, the role of the driver changes from primary to managerial control.

**Figure 2:**
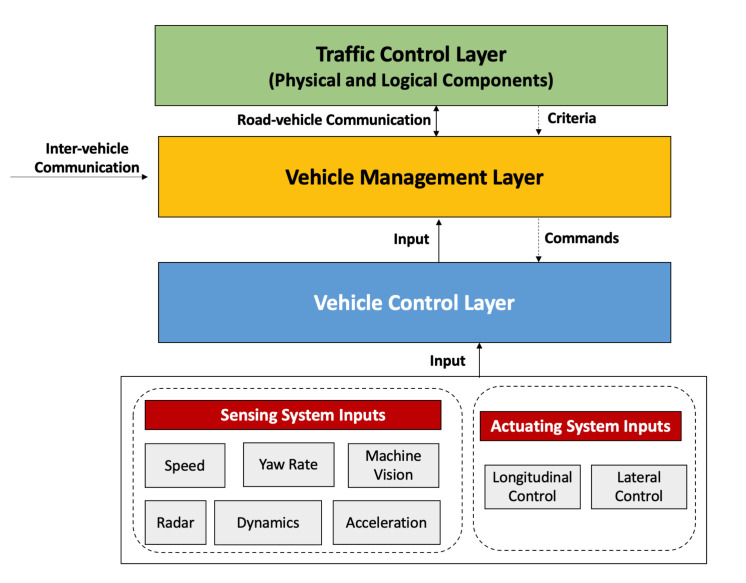
Layered architecture for CDS.

The layered architecture of CDS shown in Figure 2 is comprised of three major layers; vehicle control layer, vehicle management layer, and traffic control layer [6], where the first two layers are for each vehicle, and the third layer is common and shared by all the vehicles.

The *vehicle control layer* is responsible for two functions: (i) to detect the conditions and state variables of the following and neighboring vehicles and (ii) to activate the lateral and longitudinal actuators. This layer takes input from the sensing systems (such as various sensors for acceleration, radar, yaw rate, vehicle speed, and vision) and the actuating systems (lateral and longitudinal control), and outputs the vehicle management layer seeking to obtain steering and vehicle speed queries. The *vehicle management layer* is in charge of determining the moment of every single vehicle under the constraints of cooperative driving by utilizing the data from control layer, neighboring vehicles through IVC, and the traffic control layer through road-vehicle communications. The *traffic control layer* made up of two components: physical and logical. The physical part is composed of a traffic signal, sign-boards, and road-side communication, whereas the logical part deals with the social laws, rules, manners, and other societal ethics. Finally, this layer passes on the traffic information with each vehicle in the management layer to maintain coordination and to perform actions [2,6].

In collaborative driving, the collaboration is of three degrees [7]:

- *Longitudinal Control:* This type of automation controls the distance and velocity of the vehicle by using a cruise control system, though the driver needs to steer the vehicle manually.
- *Semi-Autonomous Control:* This type is similar to precedent one, except that both the lateral and longitudinal motion of the vehicle is autonomously regulated to the preceding it.
- *Fully Autonomous Control:* This level of autonomy allows all vehicles to be completely controlled and managed autonomously.

The elimination of the mastery entity in the fully autonomous control vehicles makes it a little complicated as it maintains and centralizes the coordination of the formation of the vehicles [8]. Moving forward to a wider level of collaboration, the vehicle platoon model uses communication technologies to coordinate the platoon members with the leader [2].

A strong motivation behind the CDS is to resolve two major problems: (1) to manage the distributed control of the vehicles and (2) to coordinate each vehicle controller’s actions.The solution to the first problem is to use longitudinal and lateral control. To resolve the second issue, the CDS assumes that the vehicles are grouped in the platoon formation. In a collaborative sort of driving, generally, a platoon is formed [8]. Concludingly, the focus of this research is on CDS related to the platoon of collaborative vehicles.

### 2.2. Aspects of Cooperative Driving

It has been pointed out that some of the underlying concepts of cooperation are not clear in the literature. Therefore, it is indispensable to understand the motivations that influence vehicles to collaborate, as well as the different kinds of collaboration types and coordination strategies. To determine this information, this sub-section discusses the motivations, followed by different collaboration types and control strategies, in detail.

**Figure 3:**
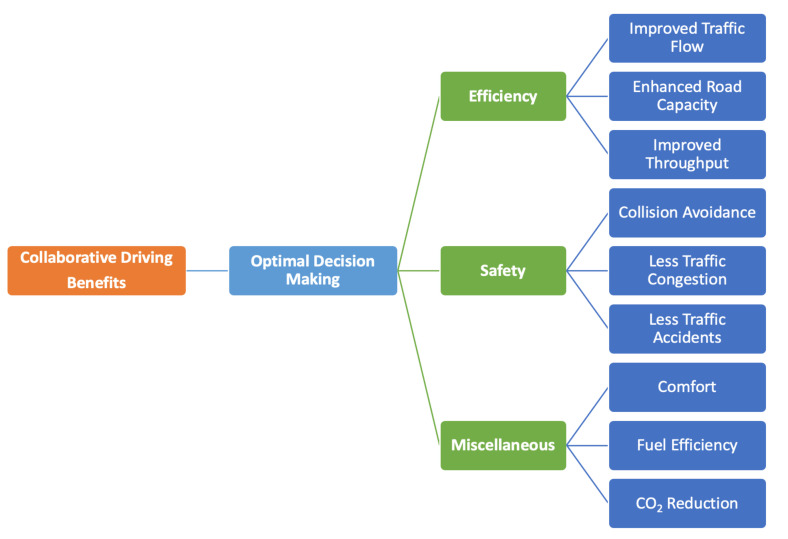
Classification of cooperative driving benefits.

1. ***Why is collaborative driving important?*** Autonomous vehicles (AVs) do not intrinsically improve traffic. They attempt to rely on their observations, perform reasoning, and make decisions aiming to optimize their own goals [9]. AVs are still limited in terms of their sensing and coordination capabilities, as their actions are dependent on the onboard sensory data and models of other vehicles’ behavior. The summary of the Urban Grand Challenge [10] stated that a number of incidents can be avoided if vehicles anticipate the behavior of other vehicles and that vehicles should cooperate to reap the full benefits of autonomous driving [11]. However, it is getting increasingly recognized that, to get full advantage of autonomous vehicles, a number of situations will compulsorily require coordinating the relative activities and maneuvers of vehicles [12].
2. ***Why should vehicular agents collaborate?*** In collaborative driving, vehicular entities cooperate, where the outcome of a vehicle’s action may influence other vehicles. Here, it is crucial to understand why a vehicle agent will collaborate with others knowingly benefitting them. Therefore, we define three motivations that influence vehicles to cooperate [13]:
  - *Mutualism:* the collaboration, where the agent interacts and cooperates with each other aiming to minimize the cost (fuel, travel time, etc.) of all agents collectively.
  - *Altruism:* the interacting agent interacts in such a way that it increases its cost while reducing the cost of other agents.
  - *Selfishness:* the type of collaboration where the agent cooperates desiring to reduce its own cost and leaving the rest in the loss.
3. ***What are the cooperation control strategies?*** To attain self-organized agent formation and to provide autonomy to each agent, we define the coordination strategy as three types [13]:
  - *Centralized Coordination:* One all-knowing, leading vehicle is responsible for the planning, coordination, and synchronization of the maneuvers of all the vehicles. The literature divides the centralized coordination strategy into two variants: leader-follower and virtual leader approaches [14].
  - *Decentralized with Coordination:* This allows the vehicles to directly communicate with all neighboring vehicles having access to local knowledge (restricted by the communication range) aiming to plan maneuvers.
  - *Decentralized without Coordination*: The vehicles can observe other vehicles in the neighborhood without having the potential to exchange the information. Each vehicle can plan individually by taking into consideration the behavior of the road users [15].
4. ***Why do we need to do collaborative driving?*** The underlying concept of vehicular cooperation is to make optimal joint driving decisions and get benefitted from the cooperation, reducing costs by mutually cooperating with each other. Having reviewed the literature, we highlight and classify the potential benefits of collaborative driving into three categories: traffic efficiency, safety, and miscellaneous, as shown in Figure 3.
5. ***What are collaboration types?*** To the best of the knowledge, there are no standard categories of collaborative driving in the literature. However, in this paper, we provide our understanding of collaboration and categorize the collaboration among the vehicular agents into three main types:
  - *Imperative Collaboration:* the type of collaboration where the agents are obligatory to cooperate with each other.
  - *Voluntary Collaboration:* the type of collaboration which makes it optional for the agents to collaborate depending on the use case scenarios.
  - *Hybrid Collaboration:* this is the combination of (imperative and voluntary) both types, which allows the agents to travel imperatively for a certain period or distance and voluntarily ends the collaboration.

## 3. Cooperative Driving—A Comprehensive Analysis of the Research Literature

It is noted that vehicular cooperation can take different forms; besides that, there is not a single correct way to implement cooperation, but there are many different options. This section focuses on analyzing the research literature on collaborative driving applications in the autonomous driving paradigm. Having analyzed the literature on generic cooperative driving applications in broader settings, we focus on a relatively less integrated and more futuristic scenario, specifically in the Society of Automotive Engineers (SAE) Level 4 (L-4) and above platooning, i.e., leader election.

Cooperative driving was first initiated using inter-vehicle communication to perform lane changing and merging maneuvers in the context of platooning [16]. Platooning has widely been studied recently ranging from communication, security, and safety, to fuel consumption perspectives. By comprehensively reviewing the literature, we conclude that very little attention has been accorded to the most important use-case of platooning; leader election.

It is needless to mention that the L-4 platooning will be a norm in urban scenarios, where the platoon formation will be more dynamic, and the leader election decisions may be triggered, with much higher frequency than the classical L-2 platooning. We believe that no survey delineates the platoon leader election and summarizes the existing solution approaches. Therefore, we make effort in this survey to focus on this issue by providing solution approaches and highlighting the future challenges.

### 3.1. Generic Cooperative Driving Scenarios

In this section, we walk through the reader with a variety of generic scenarios showing the potential of cooperative driving in addressing the on-road issues and challenges.

#### 3.1.1. Cooperative Smart Car Parking

The proliferation of vehicular traffic has made searching for a parking space a frustrating task for the driver. The INRIX research survey [17] states that American drivers spend an average of 17 h per year in finding space for parking, leading to a loss of $345 in terms of wasted time, fuel, and CO_2_ emission. Difficulties in finding a suitable parking place encountered globally result in traffic congestion and air pollution issues that need to be addressed, and urgently. Readers are highly encouraged to look into the survey conducted by Diaz et al. [18], which extensively investigated the smart parking systems and classified them into six categories: parking reservation systems, parking guidance and information systems, crowdsourcing in intelligent transportation systems, centralized assisted parking search, agent-based guiding systems, and electrical vehicle parking systems. Several other research studies have been conducted to address the car parking issues, and some of the latest solution approaches are highlighted in this section.

Correa et al. [19] implemented a smart parking system on top of the vehicular sensor network considering the heterogeneity of the cars (traditional and autonomous) in the parking slot. The proposed system communicates and assists automated vehicles in finding the best parking place based on collaborative approaches. A designed tree-based searching algorithm (TBSA) is implemented seeking to provide the best parking slot and to enhance the accessibility of the parking for other autonomous cars. The results concluded that the proposed approach can perform best when the autonomous vehicles enter the parking lot in succession. In another study [20], the authors proposed a decentralized parking approach called Cooperative Car Parking (Co-Park) leveraging V2V communication and agents installed on vehicles letting the cars find suitable parking in large areas, such as airports, urban areas, and all. The extensive simulation on the Co-Park approach by comprehensively investigating the various parking scenarios showed that the strategic cooperation among the agents leads to efficient car park searching for all.

Wu et al. [21] addressed the multi-vehicle cooperative parking issues. They implemented the model based on the kinetic model integrated with dynamics, endpoint, and collision avoidance constraints. Based on the Gauss pseudo-spectral method (GPM)-based technique, the authors transformed the planning of the trajectory of the multi-vehicle problem into an optimal control problem. The experimental results demonstrated that the GPM has the potential to effectively solve the cooperative multi-vehicle parking problems. Likewise, authors in Reference [22] focused on the planning and coordination of multi-vehicles in automated parking scenarios. This problem was modeled as symmetric mixed-integer linear optimization aimed to investigate the intentions and driving styles involved in optimization problems.

To sum up, the exhaustive survey conducted on smart parking systems (SPS) by Reference [18] uncovered several open issues and challenges in the SPS, such as:

- the consideration of user preferences while suggesting the optimally available parking spaces to the drivers;
- highlighting that the implementation of SPS should be context-sensitive since all cities have different infrastructures, budgets, and societies; and
- found that less work has been done on rescheduling the routes established by the SPS taking towards the parking slot in case of unexpected event triggers.

#### 3.1.2. Lane Change and Merge

**Figure 4:**
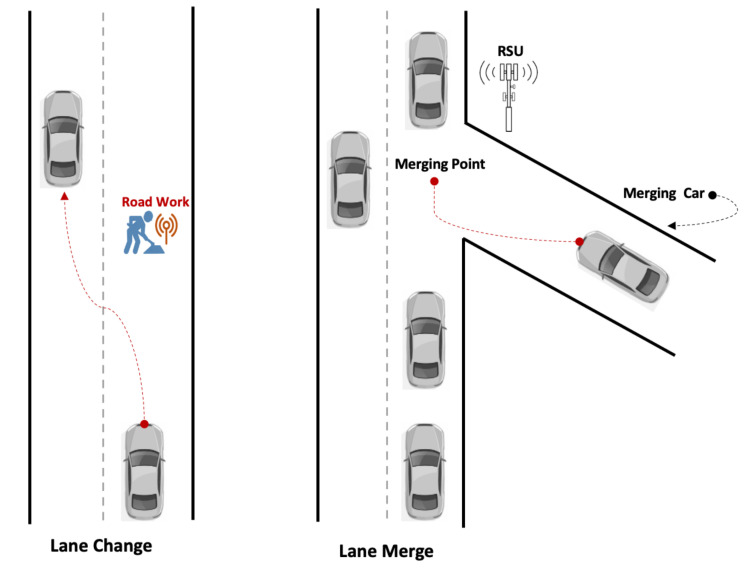
Illustration of lane change and merge.

The lane changing and merging shown in Figure 4 demonstrate one of the hazardous maneuvers that lead to one-tenth of all traffic crashes. In America, 33% of all the accidents occurred when vehicles were making a lane change or performing merge maneuvers [23,24].

Cooperative and automated lane change leveraging intelligent driving technologies can substantially enhance road efficiency and safety. The rapid growth of V2V communication has made possible the development of the cooperative automated lane-change maneuvers. Luo et al. [25] contributed to the lane change model by implementing the automated lane change maneuver using V2V communication. Considering the current state of the neighboring vehicles, the model could determine whether to carry on the lane change maneuver or to return to its original lane based on the minimum safety spacing (MSS) model. However, this model was implemented for two-lane settings and was not suitable to work with multi-vehicle lane-changing maneuvers. Predicting the state of the surrounding vehicles, a decentralized cooperative lane merging framework based on coordination strategy is developed by Reference [26] to avoid conflicts. The experimental results showed that the proposed framework could perform well in traffic dynamics. Previous V2V-based approaches did not consider the characteristics and cooperation of nearby vehicles, which results in danger while taking lane-change maneuvers. To conquer these issues, Reference [27] implemented a V2V-based cooperative lane change framework while considering the information of the surrounding vehicles to plan the trajectories. Besides, the trajectory planning is formulated as an optimization problem to increase the safety and comfort in lane changing, considering the constraints of the vehicle dynamics.

Ref. [28] exploited reinforcement learning to implement the cooperative lane change approach for connected vehicles. While setting up the reward function, the proposed study considered two factors: the delay caused by an individual vehicle and traffic efficiency of the road segment. The simulation-based results demonstrated that traffic efficiency is enhanced in congested scenarios. Given the deep learning approaches, Reference [29] implemented the hierarchical Deep Q-Network reinforcement learning-based decision-making framework for lane changing maneuver. The experimental results proved the effectiveness of the proposed architecture in terms of decision-making.

The authors of Reference [30] proposed a coordinated lane merging approach for a vehicle to everything (V2X) scenarios based on a centralized system for connected cars. The application comprised of a traffic orchestrator is developed to provide the trajectory recommendation to the vehicles. Besides, several machine learning algorithms and data analysis algorithms were implemented to determine whether the connected vehicle (CV) carried out the lane merge maneuver successfully. Ref. [31] extended this work by integrating the deep learning reinforcement learning and data analysis algorithms seeking to predict the trajectory recommendations for CV. The results concluded that Dueling Deep Q-Network outperformed the Deep Q-Network in terms of best performance and delivering human-like trajectories.

Generally, the lane merging algorithms consider the simple rules to generate the order of the vehicles for merging. To solve this problem, Reference [32] developed the cooperative merging strategy and modeled it as an optimization problem. The proposed research study applied a genetic algorithm to solve the optimization problem by taking the objective inputs of minimum travel time of vehicles and the maximum number of merging vehicles. The simulation-based experimental results showed that the cooperative lane merging approach proved the ability to improve traffic efficiency and fuel consumption. Several research studies implemented the slot-based (*slot*: the free area between two vehicles) approach to deal with the cooperative lane merging issues [33,34,35]. The aforesaid approach is comprised of two steps: (i) it determines the merging availability in the target lane; and (ii) the feasibility of actions is considered to find the optimal slot for merging acceleration. This approach makes a decision only based on current states, while ignoring the historical data, which results in failures.

#### 3.1.3. Cooperative Intersection Management

**Figure 5:**
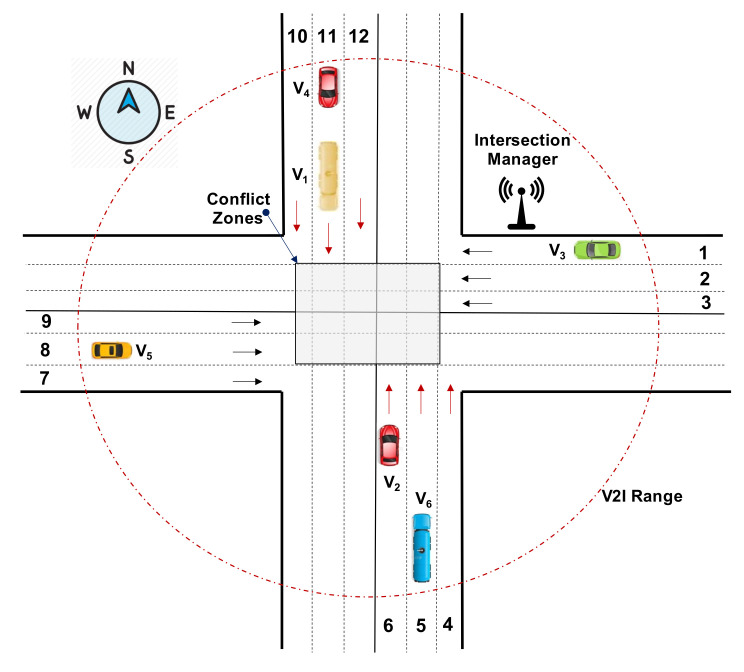
Standard 6-lane 4-leg intersection management. Source: Adapted from Reference [37].caption

Although the intersections deal with a relatively smart part or road system, the Federal Highway Administration (FHA) reports that 40% of all the accidents occurred at intersections, considering as the second-largest category of accidents [36]. Connected and autonomous vehicles (CAVs) communicate and exchange information with the Intersection Manager (IM) and with other CAVs intending to pass the intersection safely, as shown in Figure 5, whereas the conflict zone shown in the figure represents the area where the collision of the vehicles coming from different lanes can happen. Therefore, automated intersection management systems should be implemented to enhance road efficiency and safety, as well as to minimize fuel consumption and travel time.

Mainly, the intersections are of two types: signalized and non-signalized, which are controlled by V2V and vehicle to infrastructure (V2I). *Signalized* intersections have traffic lights to boost road safety and efficiency by allowing the vehicles to pass an intersection based on traffic lights, whereas *non-signalized* intersections are comprised of yield, stop signs, and sometimes with the possibility of having no signs at all. In signalized intersections, the Cooperative Intersection Management (CIM) permits the vehicles to communicate and exchange a variety of information with the infrastructure, such as traffic perception, negotiation to pass the intersection, vehicle signage, and so on, while, in non-signalized intersections, the CIM assists the drivers by providing the global view of intersection managements to make optimal decisions [38]. Some of the recent research studies on signalized and unsignalized intersection management are listed in Table 1.

The presence of traffic lights in signalized intersections has proved efficient traffic flow and safety. A considerable amount of research studies have been added to improve traffic light algorithms. Some of the well-know implemented approaches are *fuzzy logic* [39,40], *neural networks* [41,42], *mathematical models* [43], *query theory*, and *markovian-based* models.

Recent studies on a network of signalized intersections are based on max pressure and back pressure, providing algorithms that enable the smart traffic lights. The information of traffic queues and turn probabilities are the important inputs of these algorithms. In Reference [44], the authors implemented an optimization algorithm aiming to minimize the traffic queue length by giving green light to lanes having the largest queue. The traffic information was collected via V2I communications. The authors concluded that wireless communication technologies are more advantageous than the conventional loop detectors since the transmitted information is in more detail. In References [38,53], the authors implemented a V2X architecture, allowing a group of vehicles to negotiate with the controller for the green light. The vehicles estimated the traffic queue length by themselves. While awaiting the green light, vehicles communicated via V2V and grouped automatically based on their destinations. Each group elected a leader to take charge of the queue length. Finally, each leader of a group communicated their queue lengths with the intersection controller to get the green light. In another study, to avoid collision and minimize the waiting time at the intersections, Alouf et al. [45] implemented a model scheduling the AV at an intersection using production line technique and K-Nearest Neighbor algorithm to predict the right-turn of the vehicles. Their simulation-based results concluded that their model outperforms traditional models in terms of random-pattern traffic flow.

The intersections can be modeled as tiles, where the vehicles need to reserve the tiles for their decided routes for a certain time. Once the titles and time slots are approved, the vehicles can cross the intersections based on their reservation time. The authors of Reference [48] studied the cooperative resource reservation with a simple intersection layout and assumed that, once the tile is reserved, the vehicle has to maintain the same spend until the intersection is crossed. The authors compared their simulation-based experimental results with two intersection management approaches, overpass and traffic light. Results showed that their proposed outperforms overpass and was two to three times good than the traffic light method. Following this, in another study, Reference [47], the authors implemented a traffic simulator named ISR-TrafSim to test the reservation-based intersection management systems. Two types of intersections (roundabouts and crossroads) were considered, and different procedures were applied. In the case of roundabouts, vehicles sent the information to the intersection agent to notify their driving plans, whereas, in the case of crossroads, the intersection agents themselves automatically detect the driving intentions of the vehicles. In both cases, the intersection controller was responsible to allocate time slots and space tiles.

Liu et al. [37] implemented a cooperative scheduling mechanism for AV crossing the intersections. The foremost goal of their research was to minimize the delay at unsignalized intersections. Their proposed mechanism is comprised of mainly three phases: firstly, the intersection manager is used as an information collection point, and it prioritized all the vehicles and plans their trajectories. Secondly, a window searching algorithm was used to find an entering window aiming to produce collision-free trajectories. Lastly, based on the velocity profile computed by dynamic programming, each vehicle arranged itself individually to pass through the intersection. Their experimental results concluded that the proposed approach minimizes the evacuation time and improved the throughput by 20%. Similar to this study, the authors of References [49,51] worked on space and time-aware approaches to manage the AV at an intersection that is robust against external disturbances and model mismatches. They proposed that the intersection manager is responsible to assign the Time of Arrival and Velocity of Arrival to an autonomous vehicle in such a way that the route of AV before and inside the intersections does not conflict. On the other side, AV is responsible to track the best route to reach the intersection at the given Time of Arrival and Velocity of Arrival. The algorithm tested on 1/10 scale intersection of AV showed that it achieved 2.7X more throughput as compared to velocity assignment techniques.

Recently, multi-intersection traffic signal control approaches have grabbed the attention of researchers, which are more practical than single-intersection traffic signal control. Ref. [54] implemented the decentralized coordination graph algorithm based on multi-agent deep reinforcement learning and a coordination graph. The proposed algorithm is designed to make traffic control policies by exploiting the current traffic states, the history of observations, and other useful information. The experimental results conducted on real-world scenarios reported that the proposed algorithm outperformed other algorithms in terms of traveling time and delay leading to improved traffic congestion. Refs. [52,55] also implemented the Deep Q-Network (DQN)-based cooperative traffic signal control for multi-intersection. Each intersection was modeled and trained as an agent and attempted to collect the traffic information from the road environment. The extensive results showed that the proposed approach improved traffic congestion significantly at multiple intersections. Some of the other research studies worked on other approaches, i.e., centralized cooperative resource reservation, time-space reservation, trajectory planning, virtual traffic light, and intersection collision avoidance, which are extensively discussed in the studies of References [44,56,57,58].

Despite the rapid and progressive advancements in intersection management systems, we highlight some of the open issues and challenges of CIMs that need more research and advanced solution approaches to overcome these issues. Some of the common challenges are optimized resource utilization, several uncertainties from the control, communication, mechanical perspectives, vulnerable road user detection, sensing and identification of pedestrian and cyclists at non-signalized intersections, communication issues, such as delays packet loss, security, bandwidth limitations, low-quality data, distributed processing of data either at intersection controller or by CAV, and collision avoidance [46,50].

### 3.2. Vehicular Platooning

Vehicle platooning is an important use-case leading towards cooperative autonomous driving. It has obtained the significant attention of multiple stakeholders, such as researchers, truck operators, and society, because of its several potential benefits of improved road safety, less fuel consumption, CO_2_ emission reduction, road throughput, asset utilization, and labor cost optimization.

**Figure 6:**
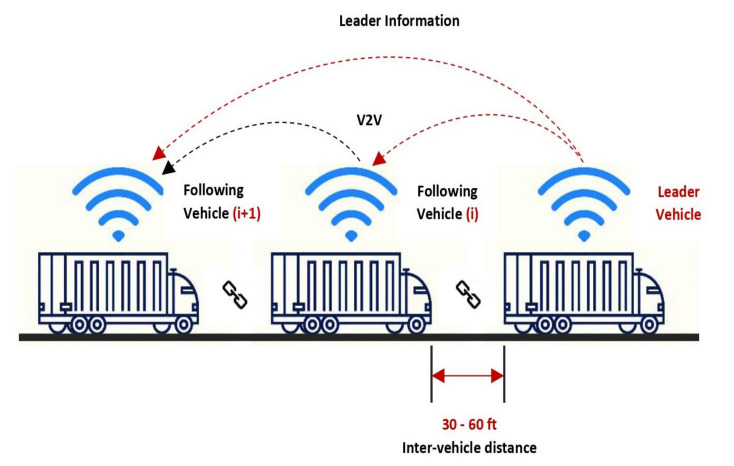
Illustration of platoon working.

Vehicle platooning, also known as convoy driving, is the practice of driving a group of two or more consecutive vehicles nose-to-tail on the same lane with small inter-vehicle spacings typically less than 1 s at the same speed. In platooning, the vehicles are linked virtually by Lidar and communicate with each other via wireless communication technologies, such as (CACC). The platoon shown in Figure 6 is comprised of two types of vehicles: the *platoon leader (PL)* and *follower vehicle (FV)*.

Usually, each following vehicle is denoted with a number, FVI, FVII, and so on, to indicate its position within the platoon. The lower number is, the more the following vehicle is closer to the leading vehicle. The leading vehicle drives at the first position and follows by a couple of follower vehicles, which means FVs can automatically brake, steer, and decelerate, depending on the actions of the leading vehicle [59]. The vehicles in the platoon coordinate their lateral and longitudinal control to maintain the required headway from their leading vehicle [60]. The leading vehicle controls the driving direction, and the FVs follow the leading vehicle using advanced technologies, such as adaptive cruise control, automated emergency braking, blind-spot warning, forward collision avoidance system, lane departure warning, etc. Once the platooning mode is activated, the following vehicles start following the leading vehicle automatically without the intervention of their drivers. The V2V communication allows the vehicles to communicate back and forth; therefore, leading vehicles can also adjust their speed and position based on the response of the FVs. The wireless coupling and decoupling in platooning make it easy for vehicles to hop-on or hop-off from the platoon on the fly without needing to stop the driving.

#### 3.2.1. Platoon Leader Election

In many applications, a leader or coordinating vehicle is required to synchronize and accomplish a cooperative goal for a group of vehicles. Some of the applications are intersection coordination, Virtual Traffic Lights (VTL) [61], and highway merging [62], and vehicle platooning is one of them. These applications allow vehicles to communicate in a V2V manner and eventually elect a leader unanimously.

**Figure 7:**
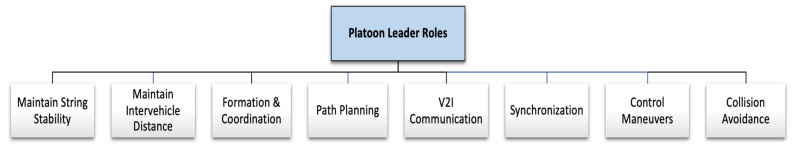
Role and responsibilities of platoon leader.

The leader vehicle plays a substantial role in platooning and responsible for the maneuvers, such as formation, merging, and splitting the platoon, by transmitting the information with the following vehicles. Some of the additional roles and responsibilities of the platoon leader are shown in Figure 7.

Besides, the leading vehicle is in charge of defining the acceleration, inter-vehicle distance, and direction for the platoon and shares this information with the trailing vehicles at a constant time interval *(t)* to maintain the string stability of the platoon [63]. In addition, it is the PL who has to be active constantly, more responsible, aware of situational conditions, control the forward traffic conditions, communicate with the infrastructure, and exchange information with the following vehicles to control and maintain the platooning successfully. Since the platoon leader takes the lead of the whole platoon, consequently, the result of its operations and decisions directly influences the safety, traffic throughput, and stability of the platoon [64]. In platooning, data transmitted by the leading vehicle to the trailing vehicle is processed by the cruise controller and directly affects the speed of the trailing vehicles. In this scenario, electing a PL with malicious intention can put the whole platoon in danger by reducing the safety [65]. Considering the primacy and manifold; responsibilities of PL, it is of utmost importance to elect the most suitable and trustworthy leader for the platoon.

In this section, six different use case scenarios are discussed where a vehicle platoon is required to make a cooperative decision to elect a leader for a group of vehicles to carry on the platooning.

1. *Electing a leader while forming the platoon for the first time:* In this scenario, while forming the platoon for the first time, a vehicle needs to be elected as a platoon leader to start the platooning. The platoon leader is then responsible for the coordination and all the operation in the platoon. Each vehicle wishing to drive in a platoon manually needs to admit it by selecting the platooning mode.
2. *Electing a leader when two platoons merge:* In this use case, two platoons of different length traveling on the same lane decides to merge and make one platoon aiming to make it more efficient to travel in the platoon. Electing a leader in the merging scenario is a challenging maneuver because of the interferences triggered by unintended vehicles that join the platoon. When two-platoon merge, it is required to re-elect a suitable leader to continue the platooning successfully. Finally, the merging platoon has to share its information with the elected leader of the platoon.
3. *Electing a leader when a platoon splits:* In the platoon splitting scenario, the platoon splits into two or more sub-platoons at a point while continuing to drive to their intended destinations. Once the platoon splitting request is acknowledged by the platoon leader, it is indispensable to elect a new leading vehicle to take in charge of leadership roles and responsibilities for the split platoon.
4. *Electing a leader while adding a new vehicle:* This scenario takes place when a new vehicle dynamically desires to become a part of the already formed platoon, driving on the road. Based on the characteristics of the new vehicle, it is recommended to take the consensus of all the vehicles and re-elect the new leader for the platoon since there is a possibility that the newly added vehicle is more suitable to lead the platoon. However, this use case could be inefficient as the vehicles have to undergo the entire process of leader election every time, which is a waste of time and resources.
5. *Electing a leader when a platoon dissolves fortuitously:* While traveling in a platoon, sometimes, due to bad situational conditions, technical issues in a leader, or momentary traffic conditions leads to platoon dissolve. At this stage, the platoon cluster is disbanded, making the complete platoon stop the platooning. In this scenario, it is essential to re-elect the leader aiming to maintain and reap the full benefits of platooning.
6. *Electing a leader when a leader leave maneuver takes place:* In this use case, the vehicle platoon is required to re-elect a leader when the *leader leave* maneuver takes place. The *leader leave* maneuver can occur in two different scenarios. The first one is when a platoon leader reaches its destination and makes the *leader leave* request, whereas, in the second case, the leader vehicle may be inoperable due to any technical failure. In both cases, the leading vehicle is incapable to perform the leader’s role and responsibilities; thus, it is mandatory to re-elect a leader to carry on the platooning.

#### 3.2.2. Solution Approaches and Challenges

The considerable amount of literature can be found on different perspectives of platooning, such as *platoon formation* [66,67], *fuel consumption* [68,69], *CO_2_ emission reduction* [70], *communication*, *string stability* [71], *control and coordination strategies* [72], and *security and safety* [73,74]. However, relatively less importance has been directed to study how to elect an experienced driver, most suitable and trustworthy candidate vehicle as a platoon leader. Therefore, a leader election problem in vehicle platooning is of great interest and needs significant research.

Vehicle platooning has been the focus of multiple stakeholders: academia and the transportation industry. In recent years, ample research has been done on the leader election problem in wireless ad hoc networks [75,76]. However, these mechanisms are not directly applicable for vehicle platooning owing to distinct objectives, mobility, and context parameters. Literature directs little consideration to the leader election problem in platooning.

**Figure 8:**
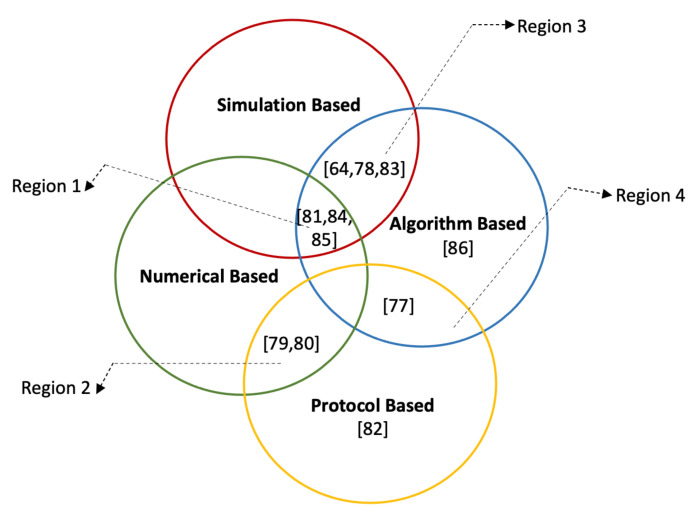
Classification of solution approaches for leader election.

In this section, we provide an exhaustive review analysis of the platoon leader election studies. Inspired by the various solution approaches implemented to elect the leader, we carefully choose a visualization approach, showing the scope of the relevant research articles in Figure 8. Besides, based on the methodologies, we classify the solutions into four broad categories. The *algorithm-based* studies are the studies where the authors developed different algorithms to elect the leader. In *protocol-based* approaches, either the authors implemented new protocols or they improved the previous one leading to elect suitable leaders, whereas, in *simulation-* and *numerical-based* approaches, authors proposed different simulations and mathematical models to elect trustworthy leaders. Finally, the studies which overlap with each other are highlighted in the *region 1,2,3* and *region 4*.

In Automated High System (AHS), usually, a special heavy vehicle is nominated as a platoon leader which restricts the election of platoon leading vehicles autonomously. To overcome this limitation, the authors proposed the Autonomous Platoon Leader Selection (APLS) mechanism [77]. The proposed mechanism exploited the mobility information: speed and velocity of neighboring vehicles to select the appropriate stable platoon leader intending to reduce the fuel consumption and traveling time of the vehicle. Based on the obtained mobility information, a suitability value *(W)* is computed for each vehicle by taking the difference of its velocity from the average velocity of all neighboring vehicles. Besides, the position of the vehicle is also taken into consideration by taking the average position of all vehicles. Eventually, the vehicle with the highest *(W)* value is selected as a platoon leader. In 2017, the authors extended this work by simulating the mechanism in the NS-3 simulator [78]. The APLS mechanism is evaluated based on three performance metrics: platoon formation message overhead, platoon stability, and platoon size. Despite the successful results, some limitations are associated with this approach. While selecting the leader, only the velocity and position of the vehicle are taken into consideration that are not good enough parameters to select a leader. Besides, the characteristics of the driver and vehicle are also not considered.

The goal of platooning is to optimize road throughput by traveling in a small inter-vehicle distance. In current vehicular platooning, if the platoon cluster splits due to technical or traffic issues, the platoon is still capable to elect a new leader to continue driving in platooning. However, this process fails to maintain the goal of maximizing road throughput. To address this problem, the SAE J2735 protocol was improved, and a new data element, the candidate vehicle is added to elect the candidate leader [79]. While joining the platoon, a vehicle has the option to set its status as a candidate leader by setting up the status value to 1. In the case of several following vehicles, wishing to be a candidate leader, the proposed approach prioritizes the candidate leader based on the status information of the vehicle. This approach made it easy to elect a candidate leader without delay based on priority information eliminating the need of going through the complete process of selecting a leader without delay. The authors did not elucidate which status information of the vehicle is modeled and how they computed this value to elect a leader. In addition, the experimental setup and results are not discussed in detail. In another research study [80], it is stated that in traditional platooning, the platoon leader is fixed and the platoon cluster has to disband in case of any uncertain situation that falls short the goals of platooning. Moreover, it is argued that it is extremely challenging to determine whether a vehicle is well enough to take the leading role of the platoon. The authors proposed a solution to this problem by applying the consensus-based Raft algorithm to automatically elect the leader in emergencies. For each vehicle, a numerical relative value is computed based on the vehicle’s performance aiming to ascertain which vehicle is capable to take the lead. After calculating the relative value, the leader is elected through the Raft algorithm. However, this study did not highlight how the numerical value is calculated and which performance metrics are taken into consideration while voting for a vehicle.

It is more advantageous traveling as a following vehicle than the leading vehicle since it is accountable for several duties and consumes more fuel [81]. The equal distribution of profit from platooning between all vehicles is crucial to collaborate and form the platoon; otherwise, competing companies will not be agreed to form a heterogeneous platoon. To mitigate this selfish behavior of users and to make it profitable for vehicles to lead, an incentivization framework should be there. Ref. [82] worked on in question problem and proposed a protocol named Leadership Incentives for Platoon (LIPs) for dynamic and heterogeneous platoons. A comprehensive incentivization system based on blockchain technology is implemented to provide near-equal benefits to the platoon leader. Several parameters (fuel price, distance, average savings of followers, etc.) and service charges are modeled to determine how much money a vehicle should pay to a leader to travel as the following vehicle in the platoon. However, this approach neglected the leader election issue and did not consider the position of vehicles in the platoon to compute the accurate monetary cost. Similarly, in another study [83], an urban platoon-driving model is developed to allow vehicles to choose the best path-matching platoons. Additionally, a platoon head rotational selection mechanism and an incentivization framework based on blockchain technology are implemented to incentivize the leaders. A rule is formulated to select the most credible and trustworthy vehicle as a platoon leader by calculating the reputation score of vehicles. Each elected platoon leader is required to drive a certain distance and receive service charges from the following vehicles. Finally, to incentivize the leader, the fuel consumption and services charges are modeled. In the proposed study, it is not discussed how the cost of fuel consumption is calculated and whether the simulated platoon is homogeneous or heterogeneous. The downsides of References [82,83] is that both studies are the introduction of the complete transaction system that increases the cost, and the estimation of the cost of fuel consumption is a non-trivial problem [84]. In 2018, Reference [64] also worked on a similar problem to elect a leader based on an incentivization strategy. A round-based consensus 1-of-n selection algorithm is used to elect a leader. To incentivize the platoon leader, they added the third party, the insurance company, to provide the subsidy to the leading vehicle on its next insurance premium. This study is merely a proposed idea and suggested that vehicular characteristics and driver rating information should also be considered while selecting a leader.

A malevolent elected leader can put the complete platoon into danger; therefore, it is important to select an honest, trusted, and reputable vehicle to accomplish leading roles. Ref. [85] utilized the blockchain at the roadside unit (RSU) and the platoon level to elect a trustworthy leader. Based on the reputation score, each vehicle voted for other vehicles consequently, a vehicle with the highest votes elected as a leader. Ref. [86] implemented the reliable trust-based platoon service recommendation scheme (REPLACE), assisting users to not elect a malicious badly-behaved platoon leader. Besides, a reputation score system, based on the vehicle’s historical performance feedback, is designed for the platoon leader to distinguish between the well-behaved and badly behaved vehicles.

To sum up the analysis, we uncover the several limitations of the current research studies:

- Most of the studies did not model the characteristics of the driver and vehicle while electing a leader.
- Less attention has been directed on how to re-elect a leader when the cluster disbands in an emergency or at traffic signals.
- Fewer studies focused on electing a leader in heterogeneous platoons.
- It has not yet been studied whether being a leader is more beneficial in homogeneous or heterogeneous platooning.
- The voting criteria or parameters to elect a leader need to be researched in detail.
- Some of the studies implemented voting mechanisms to elect a leader; however, the comparison of these mechanisms is missing in the literature.
- Several studies did not consider the position of the vehicle while calculating the money for fuel consumption that a follower has to pay to the leader.
- More work needs to be done on the incentivization frameworks and equal distribution of the profit gained by the platooning.

Having analyzed the literature review on the platoon leader election, in this section, we highlight and classify some of the trivial and still open research challenges that need to be taken into consideration while selecting a leader. Both academia and industry are looking into knitting the enabling technologies to reap the full benefits of platooning. A few obvious research questions may be formulated in this regard, such as:

1. **Homogeneous and Heterogeneous**
  - **Question 1:** Can a standardized platform be developed to choose a leader in homogeneous platooning, where all the vehicles can meet the leadership criteria? Do we need to choose the leader wisely if the vehicles are of the same fleet operators?
  - **Question 2:** In the near future, platoons will be of the mixed string of heavy motor vehicle (truck) and light motor vehicle (car). Which vehicle should be elected as the leading vehicle of the platoon? How can an appropriate platoon leader election take place in the string of mixed vehicles, considering all the communication and contextual requirements?
  - **Question 3:** Can real-life platooning be implemented as multi-brand platooning? If so, can we develop a universal mechanism to elect a suitable platoon leader in multi-brand?
  - **Question 4:** Would the multi-brands get agree to cooperate and exchange their private and confidential information with each other to elect a leader on the fly?
2. **Communication**
  - **Question 1:** How can the string stability of the platoon be maintained when the number of trailing vehicles increases? Do we need to look for new wireless communication technologies to overcome this issue? Or can this challenge be addressed by nominating a virtual platoon leader aiming to provide seamless and reliable communication to a long platoon?
  - **Question 2:** Which characteristics and parameters should be taken into consideration to select an intermediate virtual leader for a long platoon?
3. **Modeling Driver and Vehicle’s Characteristics**
  - **Question 1:** How can we model and rate the characteristics of the driver (age, behavior, mental stability, driving experience, trips, platooning training, traffic violation, and accident record) while selecting an appropriate leader? Do we need to model the characteristics of the leading vehicle driver, as it is expected that, in the near future, vehicular platooning will completely be driver-less?
  - **Question 2:** How can we develop a system good enough to model the characteristics of the vehicle (fuel quantity, tire pressure, mileage, and insurance status) while electing the leader? Do these characteristics have an impact on the suitability of the leading vehicle election?
4. **Trustworthiness and Equal Profit Distribution**
  - **Question 1:** One malicious badly-behaved platoon leader can lead the complete platoon into danger. What are the crucial factors that should be taken into consideration to determine the trustworthiness of the platoon leader?
  - **Question 2:** To mitigate the effects of trailing vehicles acting selfishly and to promote the platooning widely, how can we ensure the fairness and equal distribution of the platooning benefits for the leader?

## 4. Challenges and Future Recommendations

The survey conducted in this paper revealed many interesting aspects related to the research gap, challenges, and future directions for cooperative driving, as discussed below.

- **C1: Cooperative Decision-Making.** The majority of the current literature focuses on the vehicles that are already collaborating or determine how they should behave cooperatively in a particular scenario [68]. However, less attention has been directed to propose solutions that assist vehicles to decide when and where a vehicle should collaborate [87]. Therefore, a decision-making factor is missing in current solution approaches. Considering this research gap, we spotlight some of the decision-making challenges as follows: When it is beneficial for a vehicle to collaborate or travel solely? With whom should a vehicle collaborate? Which factors should a vehicle take into account to make the cooperative decision? Do the external factors (i.e., weather, road topography, mixed traffic) have an impact on decision-making? How long should a cooperation be continued to maintain the string stability of the group of vehicles? It is also interesting to research that whether the benefits of cooperation should be available to the vehicles in advance and whether they will be more selfish or cooperative if the incentives are known in advance.
- **C2: Designing Generic Cooperative Frameworks.** Prominent work has been done on developing the solution approaches for various single test scenarios (i.e., platooning, intersection crossing, lane change and merge) of collaborative driving guiding vehicles on how to behave cooperatively in the given situations. The limitation of these solution approaches is that they work well for the scenarios for which they are developed, thus providing no general suggestions in other scenarios [1,88]. For example, an algorithm developed for cooperative lane change directs the vehicles on how to change the lane cooperatively, but, at the same time, the algorithm provides no recommendations when the vehicles cross the intersection. The road traffic involves a large number of different scenarios, which cannot possibly be handled by a particular single approach. Therefore, it is of great significance to develop a generic solution approach for collaborative driving such that it is applicable for various cooperative applications incorporating a variety of objectives. Thus, developing such frameworks will allow the vehicles to adapt cooperative behavior in any situation.
- **C3: Multi-objective Optimization.** Although cooperating driving would become the necessity of autonomous vehicles soon, it is indispensable to allow cooperating agents to take their views (goals, costs, and constraints) into account to decide whether to perform a cooperative maneuver or reject a cooperation request. Mainly, existing approaches for cooperative driving have in common that they only consider global objectives, such as collision avoidance or traffic efficiency, and they do not take into account the local objectives: individual preferences, objectives, and constraints of agents. The underlying concept of vehicular cooperation is to reduce costs by mutually cooperating, but, if this is not the case, then the collaboration may result in increased cost for some of the vehicles. Hence, there is a strong need to develop multi-objective frameworks providing both (local and global) objectives to incentivize the vehicles and promote cooperative driving.
- **C4: Scale of Collaboration.** There is less chance that all the vehicles traveling in the country or city collaborate with others. Vehicles usually seek collaborations in their vicinity. A few research questions need to investigate regarding the scale of cooperation, such as: What should be the minimum number of the vehicle to achieve the potential benefits of collaboration? Can the vehicles collaborate at both local (i.e., certain kilometers) and global levels (i.e., district level)? How to predict the benefits of collaboration on a larger scale?
- **C5: Heterogeneous Cooperation.** In reality, the vehicular traffic is of mixed string: heterogeneous (i.e., heavy-duty vehicle, car) and multi-brand (i.e., BMW, Tesla) autonomous vehicles. These vehicles may have different constraints including sensors, perception, communication topologies, communication delay, spacing policies, feedback controllers, and vehicle lag. Considering all these differences, it is extremely challenging to develop the standard system in such a way that enables heterogeneous vehicles to cooperate with each other. Another challenging aspect of heterogeneous cooperation is whether the multi-brands agree to cooperate and share information with their competitors.
- **C6: Information Sharing and Data Format.** Vehicles share their information (position, speed, location, velocity, etc.) with other neighboring vehicles. Each vehicle creates a picture of the other vehicles and reacts to this information to enhance the situational-awareness and to cooperate. Having reviewed the literature, we highlight some of the open research challenges that need more attention: Which information should vehicles share to cooperate? Do vehicles share similar information in all cooperative scenarios? How often should they disseminate their information? How do vehicles share their knowledge, perception, and intentions with others? In which data format is the information shared? Does the sending agent pre-process the information itself and forward the output or does the receiving agent have to do it? In general, developing a coherent model of the surroundings of the vehicle by aggregating and fusing information from diverse sources is a major research challenge.
- **C7: Communication and Control Strategy.** In this section, we highlight some of the unanswered communication and control challenges, such as: A lot of research has been done on collaborative driving using both centralized and decentralized control, but it is still not clear which control strategy is best in each cooperative scenario. It is interesting to research whether a system can be developed with hybrid control strategies.
- **C8: Mixed Traffic.** It will take significant time to completely replace the on-road manual vehicle. Less focus has been accorded to mixed traffic settings while modeling the problem assuming negligible interaction with manual vehicles. Now, the related challenges are: How will CAVs co-exist with other manual vehicles? Do we need to create separate markings and lanes for cooperative and manual vehicles? The mixed traffic is unpredictable; therefore, is it important to research to what extent mixed traffic can affect cooperative driving?
- **C9: Quality of Cooperation.** To ensure the full benefits of cooperating driving, it is important to determine whether the cooperative action or maneuver is cooperative or not. Literature directs less attention on how to measure and rate the quality of cooperation; therefore, some research gaps are highlighted as follows: What are the different parameters that a model needs to be considered to measure the quality? Should the quality of cooperation be modeled as a qualitative or quantitative measure? Does the quality should only concerned with objectives or the reaction time of the vehicle, as well? Who should keep the record of the quality? The individual vehicle, city authorities, or the central controller? Concludingly, we suggest that the cooperative ranking system should be developed enabling vehicles to rank their cooperative experience so that, next time, vehicles can use previous actions to make a new decision.
- **C10: Selfish Behavior and Deception Detection.** Trusted communications play a crucial role in promoting cooperative driving. In CD, sometimes vehicles may behave selfishly and cooperate desiring to reduce their own cost and leaving the rest in the loss. These selfish agents are referred to as *free-riders* which utilize the external information provided by other agents to get benefit from the cooperation but do not share their exploration and observations with other agents. Here, the question is should that vehicle be banned for future cooperations? Or do we need to maintain the record of such vehicles to avoid selfish behavior in the future? Therefore, more sophisticated algorithms need to be developed to detect selfish behavior. Vehicles share their information (speed, location, etc.) and observations with other agents, determining the information is authentic or not is another major challenge in cooperative driving. Here, some questions arise regarding the information authentication, such as: (1) Who is responsible for information authentication (each vehicle or central controller) before forwarding it to others? And (2) what if a false message is communicated among the coalition? How quickly can updated information be sent to them to stop reacting immediately?
- **C11: Standards, Law and Rules.** Achieving cooperative driving requires vehicles to speak the same language aiming to share information and make joint decisions. However, there is no standard protocol to disseminate the decision-making information among agents to achieve cooperative driving. Therefore, it is all-important to develop standard cooperative protocols so that each vehicle can coordinate and cooperate with other vehicles to increase the reaction time, making the driving environment more safer and comfortable. There could be a lot of cooperative protocols, such as: for platooning, car parking, lane changing, lane merging, overtaking, etc. Now, the challenge is how to develop the mechanism enabling cooperative vehicles to trigger the selection and prioritize the right cooperation protocol under certain dynamics or while switching between the cooperative protocols. Some other challenges in this regard are the traffic laws implemented in the countries, for example, the specific speed limit in the urban and rural areas. In addition, in some countries, there is a rule that vehicles should make way for the emergency vehicles (ambulance) and government vehicles. Here, the challenge is how to program the connected vehicles in a way that they respond to these uncertain situations safely.

## 5. Conclusions

In this paper, we have elaborated on the concept and significance of one of the autonomous driving decision paradigm known as cooperative driving. To equip the reader with background knowledge, the detailed taxonomy of collaborative driving and its important aspects are also discussed in detail. The paper provides a comprehensive literature review on various generic scenarios of cooperative driving while focusing on the leader election use-case of platooning. In addition, we highlight and classify some of the important leader election challenges that need to be addressed while selecting an appropriate leader in high-level platooning. Finally, we provide a wide range of future challenges and recommendations for cooperative driving to motivate more innovative research in this area.

## Figures and Tables

**Table 1:** Summary of literature review on cooperative intersection management.

Intersection Type	Research Studies
Signalized	[38,44,45,46,47,48]
Non-Signalized	[37,49,50,51,52,53]

## Data Availability

Not applicable.

## Abbreviations

The following abbreviations are used in this manuscript:

ACC	Adaptive Cruise Control
AHS	Automated Highway System
AV	Autonomous Vehicle
CACC	Cooperative Adaptive Cruise Control
CAV	Connected and Autonomous Vehicle
CC	Cruise Control
CD	Cooperative Driving
CDS	Collaborative Driving System
CIM	Cooperative Intersection Management
CO_2_	Carbon Dioxide
CV	Connected Vehicle
DQN	Deep Q-Network
FHA	Federal Highway Administration
FV	Follower Vehicle
GPM	Gauss Pseudo-Spectral
IM	Intersection Management
ITS	Intelligent Transportation System
IVC	Inter-Vehicle Communication
MSS	Minimum Safety Spacing
PL	Platoon Leader
RSU	Road Side Unit
SAE	Society of Automotive Engineers
SPS	Smart Parking System
TBSA	Tree-Based Searching Algorithm
V2I	Vehicle to Infrastructure
V2V	Vehicle to Vehicle
V2X	Vehicle to Everything
VTL	Virtual Traffic Light
